# Influence Mechanism and Optimization Analysis of Technological Parameters for the Composite Prepreg Tape Winding Process

**DOI:** 10.3390/polym12081843

**Published:** 2020-08-17

**Authors:** Bo Deng, Yaoyao Shi, Tao Yu, Pan Zhao

**Affiliations:** 1 Laboratory of Aero-engine High Performance Manufacturing, School of Mechanical Engineering, Northwestern Polytechnical University, Xi’an 710072, China; shiyy@nwpu.edu.cn (Y.S.); pan.zhao@nwpu.edu.cn (P.Z.); 2 Xi’an Electronic Engineering Research Institute, Xi’an 710100, China; scor00@163.com

**Keywords:** composite tape winding process, fiber reinforced polymer composites, coupling mechanism, parameters optimization, response surface methodology, desirability function

## Abstract

Composite prepreg tape winding technology has proven to be an effective method for manufacturing revolving body composite structures in aerospace field. Process parameters including heating temperature, tape tension and roller pressure have an important impact on the winding products’ mechanical property such as tensile strength. The aim of this study is to investigate the influence mechanism and optimization analysis of parameters for the composite prepreg tape winding process. Firstly, the sensitivity analysis for single parameter had be employed to reveal the influence mechanism of each winding parameter change on tensile strength. Secondly, iso-surfaces analysis for parameter range had be applied to describe the distribution law of parameter with continuous distribution characteristics. Then the coupling analysis for process parameters was carried out employing response surface methodology. The analysis results showed that tape tension has the most significant effect on the winding products’ tensile strength. And the outstanding parameter combination with the heating temperature of 72 °C, tape tension of 307 N and roller pressure of 1263 N was provided by response surface design software via desirability function method. The validation experiments showed that the optimal parameter combination has a positive guiding significance for improving the quality of winding products.

## 1. Introduction

Composite materials have been widely used in engineering applications given their high specific intensity and specific rigidity [1,2,3,4,5,6]. Composite prepreg tape winding—a process utilized to fabricate revolving body composite structures—is recognized as an effective method for producing aerospace products, such as solid rocket motor nozzles, satellite fairings and missile noses [7,8,9]. The winding process can be described as the prepreg tapes’ hoop winding process designed to fabricate the rotary body composite [10,11]. Under the pressure and heating of compaction roller, the tensioned prepreg tapes are steadily wound around the mandrel rotating at a constant speed. The wound product will be cured and demolded to become the final product. Studies have shown that the process parameters during the winding process, such as heating temperature, tape tension and roller pressure and winding speed have a significant effect on the mechanical property of the composite wound products [12,13,14,15,16,17]. As an important index to characterize the mechanical properties of wound products, hoop tensile strength is employed to reflect the ability of composite cylindrical parts to resist hoop tensile failure. To improve the hoop tensile strength of the wound products, the study of the influence mechanism and optimization analysis of technological parameters in the composite prepreg tape winding process is necessary.

In recent years, more and more scholars have made great contributions to the research of composite winding technology. Some new filament winding technologies and equipment have been appeared as the technology develops. For example, Sorrentino et al. [18] used the robotic filament winding to manufacture a complex shape structural part belonging to aeronautic field. Libonati et al. [19] provided a modified classic filament winding named squeeze-winding to fabricate the bone-like structure characterized by axial cylindrical features. With the advantages of scouring resistance and high temperature resistance, the fiber-reinforcement composite tape winding products are usually used for aerospace vehicle. Therefore, the physical or mechanical properties of the winding products need to be carefully studied. Such as Wang et al. [20] researched the void growth and fiber volume fraction in the filament winding process. And Eggers et al. [21] evaluated the mechanical response of filament winding composite rings under tension and compression. Moreover, the process parameters play an indispensable important role in improving the winding products’ various performances. In terms of process controlling, the control strategies for winding tension [22] and compaction pressure [23] are the main object of study. Some scholars have studied the influence of process parameter on the properties of an end winding product. According to Mazumdar et al. [12] and Mack et al. [13], several parameters such as heat intensity, winding velocity, roving tension, consolidation pressure, of tape and tool material and cooling rate can affect the properties of an end product. In Nath’s article [14], four control factors machine speed, roller pressure, tape tension and tape temperature were investigated for the tape winding process. Meanwhile, processing temperature, winding speed and compaction pressure were selected as the key parameters to study for tape winding process according to Dai et al. [15] and Zaami et al. [16]. However, three parameters including tape tension, roller pressure and temperature were analyzed for developing a NC tape winding machine from a research of Shi et al. [17]. In order to save experimental resources unfortunately, three main process parameters tape tension, roller pressure and heating temperature are considered in this manuscript. At last, a reasonable parameters combination will contribute to improving the performances of winding product. For the parameter optimization method, Nath [14] used Taguchi’s robust design methodology to optimize the winding parameters to enhance the performance of solid rocket nozzle. For another example, Colombo et al. [24] presented an analytical study for the optimization of the filament winding parameters of a plain pipe in glass reinforced polymers composite.

According to the multitudinous researches, we can clearly understand that parameter influencing, process controlling and parameter optimizing on the winding process have been widely studied. These scientific research results have actively promoted the process development and industrial application of composite winding technology. However, the influence mechanism of single factor, multi-factor coupling and parameter range of the process parameters in the composite prepreg tape winding process on the winding products lack a systematic summary.

The aim of this study is to investigate the influence mechanism and optimization analysis of parameters for the composite prepreg tape winding process. First of all, heating temperature, tape tension and roller pressure are selected as the key important process parameters. And then the hoop tensile strength will be selected as the mechanical property of winding products.

In our engineering practice, we devoted to developing a quality control system for the prepreg tape winding products. For a particular prepreg tape, we need to give a suggested parameter combination via theoretical model, empirical model and experimental verification. And in the actual manufacturing process, the types of prepreg tapes are usually limited. Our work in this article will provide an analysis process for how we studied and obtained the parameter combination.

At first, we intended to research importance degree and distribution characteristics of the single parameter in the winding process model. According to the studies of Nikishova et al. [25] and Saltelli et al. [26], sensitivity analysis can be used to identify the key parameters which affect model performance. It plays an important role in model parameterization, calibration, optimization and uncertainty quantification [27]. The iso-surface method is a method of computer graphics. In three-dimensional space, a set of curved surface graphs are fitted by points with the same magnitude in a certain physical quantity. Then the curved surface can be applied to describe the distribution law of those physical quantities with continuous distribution characteristics [28,29]. In the paper, sensitivity analysis for single parameter will be applied to research the influence mechanism of each winding parameter change on the tensile strength. Then the iso-surfaces analysis for parameter range will be applied to describe the distribution law of parameters with continuous distribution characteristics.

Then response surface methodology will be employed to carry out the coupling analysis for process parameters. The response surface methodology, as a collection of mathematical and statistical techniques, has been widely used to develop meta-models due to its low computational effort [30]. The method is effective for developing, optimizing and improving productive processes when many factors affect one or more particular response values [31,32].

In addition, the desirability function method has been widely applied to the multiple response optimization process for the advantage of simplicity [33,34,35]. The desirability function method can convert each response variable to an individual desirability function [33]. Here, we want to obtain the optimal parameter combination by the aid of the response surface design software based on desirability function method. At last, the authors wish these researches will be useful to enhance the winding products’ quality in the composite tape winding manufacturing process.

## 2. Composite Prepreg Tape Winding Process

**Figure 1:**
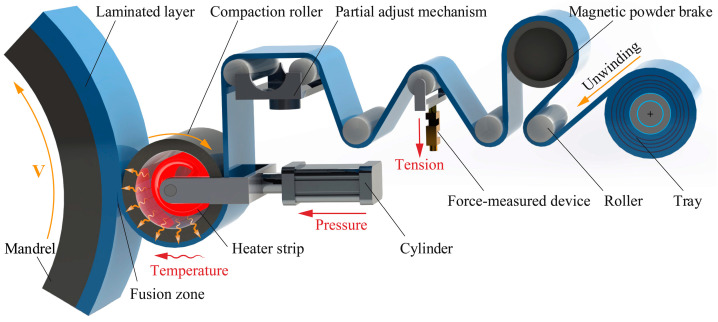
Schematic of composites prepreg tape winding process.

Composite prepreg tape winding technology can be defined as a composite fabrication process by which resin-impregnated tapes are consolidated around a rotating mandrel shape with a geometric path and tension to create a structural solid of revolution [7,36]. Figure 1 gives a three-dimensional schematic of the composites prepreg tape winding process. In the tape winding process, the prepreg tapes are pulled and placed on a series of specific rollers from the tray as shown in Figure 1. Generally, the material tray will provide the prepreg tape with a certain pretension to ensure the smooth conveyance. At the same time, the magnetic powder brake provides the required tensile tension for the tape during the winding process. The force sensor will monitor the tension value in real time and feed back to the control system. Meanwhile, we can see that the high precision partial adjust mechanism has been adopted to maintain the correct winding trajectory instead of deviation. At first, the unwinding tapes need to be heated to the suitable temperature via the electric heating device from the inside of compaction roller. At the same time, the cylinder provided compaction roller pressure on the surface of tape. Subsequently, the unwinding prepreg tapes can be continuously winded to the outer layers of the mandrel with an invariable speed. During the composite tape winding process, the heating temperature will contribute to reducing the resin viscosity and improving the degree of interlaminar contact and positive force provided by a compaction roller is conducive to squeezing out the bubbles among the interlaminar contact interfaces and enhancing the contact degree of interlayers. In addition, a suitable tape tension can greatly strengthen the hoop tensile strength of tape winding products [36,37].

## 3. Experimental Design and Procedure

Tensile strength is a representative mechanical property index for the composite tape winding products. As the composite tape winding is a complicated process, an ideal theory model describing the objective law between winding process and technological parameters cannot be easily established. In this work, we were devoted to building a mathematical simulation between parameters and responses via response surface methodology. However, the simple linear functions are difficult to explain the behavior of the complex system. To reduce the cost of computing, in practice, we choose to select the second order polynomial regression to simulate and analyze the composite tape winding process.

In this paper, a second order polynomial regression model was employed to describe the changing rule of tensile strength with the process parameter. The tape winding process model of the influence mechanism of technology parameters on the tensile strength can be presented:

(1) $$T_{S}=f\left(x_{1},x_{2},\ldots ,x_{n}\right)=\alpha_{0}+\overset{n}{\underset{i=0}{\sum }}\alpha_{i}x_{i}+\overset{n}{\underset{i=0,j=0}{\sum }}\alpha_{ij}x_{i}x_{j}+\overset{n}{\underset{i=0}{\sum }}\alpha_{ii}x_{i}^{2}+\varepsilon$$

where *T_S_* is the tensile strength of winding product, *n* is the number of variables, *x_i_* is the *i*th winding process parameter; *α*_0_ is the constant term, *α_i_* represents the coefficients of the linear parameters, *α_ij_* represents the coefficients of the interaction parameters and *ε* is the residual associated with the experiments.

### 3.1. Experimental Design

The response surface methodology is generally applied to experimental situations where several independent variables influence a response variable [34]. As a type of response surface methodology, the Box–Behnken design is rotatable second-order designs based on three-level incomplete factorial designs, allowing the number of design points to increase at the same rate as the number of polynomial coefficients [34,38]. In this study, the Box–Behnken design was chosen to find out the relationship between the response functions and variables [39]. Therefore, the three-factor and three-level Box–Behnken design based on response surface methodology theory was used to design this multi-factor and single-target experimental process.

In the experiments, the range of heating temperature *T* is between 40 and 100 °C, the tape tension *F* is between 100 and 500 N and the roller pressure *P* is between 500 and 2000 N. Meanwhile, the interval of process parameters in this paper came from an approximate range of experience and manufacture practice. To facilitate the latter part of comparative analysis process, we used a formula to converted each parameter into a coding system in the first place. Assuming that *x*_1_, *x*_2_ and *x*_3_ denotes the temperature, tension and pressure, respectively, and that *β_i_*_1_, *β_i_*_0_ and *β_i_*_−1_ represent the variable levels 1, 0 and −1 for each process parameter, respectively. Then the three variables can be described:

(2) $$x_{i}=\frac{\beta_{i}{}_{j}-\beta_{i0}}{\Delta_{i}}\;\;\;\left(i=1,2,3;\;\;\;\;j=-1,0,1\right)$$

where *x_i_* is the code of the *i*th variable, *β_ij_* denotes the *j*th variable level of the *i*th processing parameter, *β_i_*_0_ represents the 0 level of each processing parameter. Δ*_i_* represents the interval range of the *i*th processing parameter, which can be calculated as Δ*_i_* = (*β_i_*_1_ − *β_i_*_0_).

The test levels and coding results of processing parameters were combined and displayed in Table 1. The experiments were performed 17 times based on different parameters’ combinations.

### 3.2. Sample Preparation and Measurement

In the experiments, all the test specimens were fabricated on the Automate Tape Winding KUKA Robot (XGD-1200) (KUKA, Augsburg, Germany) independently designed and manufactured by Northwestern Polytechnical University, China. The compaction roller was made of 45 steel with the size of 160 mm in external diameter and 150 mm in width. The winding mandrel was a 45 steel cylinder with a size of 150 mm in external diameter and 1200 mm in length. In addition, a high precision partial adjust mechanism has been adopted to maintain the correct winding trajectory instead of deviation.

The materials used in the tests were carbon fiber-reinforced epoxy resin unidirectional prepreg tapes. The carbon fiber and epoxy resin were T-300 coming from Toray Industries, Inc. (Tokyo, Japan) and YH-69 provided by Wuxi Resin Factory of Bluestar New Chemical Materials Co., Ltd. (Wuxi, China), respectively. The width of the prepreg tape was 50 mm and the thickness was 0.2 mm.

All the winding specimens were manufactured by way of hoop winding. During the whole winding process, the ambient temperature was 20 ± 2 °C. And the mandrel rotated steadily at a speed of 10 revolutions per minute from beginning to end. Finally, the winding products were cured in the autoclave manufactured by TEDA Industrial Equipment Co., Ltd. (Tianjin, China). Meanwhile, the heating rate in the autoclave was strictly controlled at 2.5 °C/min. In addition, the curing temperature need to be consistently retained for 120 min after the temperature rose from room temperature to 150 °C. At the same time, the curing pressure must be kept around 0.15 MPa.

**Figure 2:**
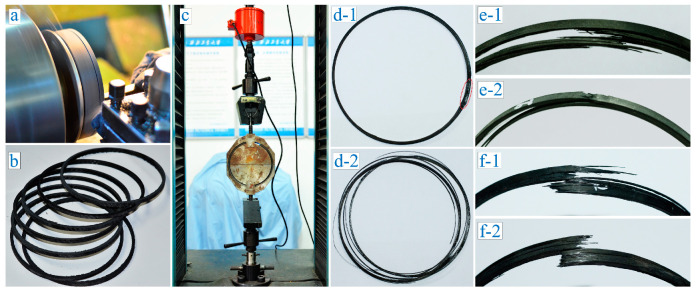
Experimental test samples machining and tensile strength testing process. (**a**) Turning machining process; (**b**) Standard testing rings; (**c**) Split disc tensile tests on electronic universal testing machine DDL100; (**d**–**f**) Typical failure mode of the ring specimen.

Since we had obtained the cured winding products, a split disc test method was developed for the determination of the apparent hoop tensile strength of composite winding ring-shaped specimens. As shown in Figure 2a, the standard testing rings for split disc tensile tests were turned along the tape winding direction on the universal lathe. Figure 2b displays the standard testing rings which have a same dimension of 150 ± 0.2 mm in inner diameter, 6 ± 0.2 mm in width and 3 ± 0.1 mm in thickness. The measurement for the hoop tensile strength of tape winding specimens were carried out following the standard GB/T 1458-2008 [40]. All the tensile strength tests were accomplished on the electronic universal testing machine DDL100 (Figure 2c) produced by Changchun Research Institute for Mechanical Science Co., Ltd (Changchun, China). According to the standard, the computational formula for the hoop tensile strength of composite tape winding ring can be given as

(3) $$\sigma_{t}=\frac{F_{b}}{2b\cdot h}$$

where *σ_t_* denotes the tensile strength *T_S_*; *F_b_* means the maximum load and *b* and *h* are the width and thickness of specimens, respectively.

### 3.3. Experimental Results and Analysis

Based on Table 1, the turning test was carried out 17 times. Figure 2d–f shows the typical images specimens after tensile failure. From overall view of standard testing ring, some fractures occurred locally just as the red box selection in Figure 2d-1. This means that a considerable weak part exists in the wound ring. By contrast, some specimens had a global decomposition-type failure mode as shown in Figure 2d-2. This is probably because the adhesive force between the fiber and the substrate in the corresponding specimen was relatively unsubstantial. As a result, more dispersion than fracture occurred among the fibers of specimen. In terms of the split form of the fiber bundle, a part of testing rings as shown in Figure 2e-1 had cracks along the ring direction and breakages in different locations. However, another part as shown in Figure 2e-2 had cracks in the radial direction and breakages in different positions. From the basic shape of the crack, some fractures were irregular and destroyed in an interlaced manner in Figure 2f-1. Still, some specimens’ fractures look comparatively flatter than the irregular ones as shown in Figure 2f-2.

All the test results of tensile strength for standard testing winding rings were recorded in Table 2. In the table, *T* is the heating temperature, *F* is the tape tension and *P* is the roller pressure. From the table, the maximum and minimum values of tensile strength are 1124.31 and 721.39 MPa, respectively.

The experimental factors and tensile strength were converted into matrix form by the test parameter transformation. The coefficients of Equation (1) were obtained from the Design Expert 10 (Stat-Ease, Inc., Minneapolis, MN, USA). Then the polynomial regression model of the influence mechanism of technology parameters on the tensile strength is given as follows:

(4) $$\begin{array}{cc} Y_{TS}=f\left(x_{1},x_{2},x_{3}\right) & =1121.008+10.31\cdot x_{1}+12.488\cdot x_{2}+8.758\cdot x_{3}+21.555\cdot x_{1}\cdot x_{2}-26.980\\ & \cdot x_{1}\cdot x_{3}-96.525\cdot x_{2}\cdot x_{3}-71.604\cdot x_{1}{}^{2}-182.739\cdot x_{2}{}^{2}-101.6889\cdot x_{3}{}^{2} \end{array}$$

To find out the significant influencing factors and reveal the interactions between the factors, the analysis of variance for samples was carried out based on the response surface design software Design Expert 10 (Stat-Ease, Inc., Minneapolis, MN, USA). The analysis results are shown in the Table 3. According to the analysis of variance, the *F* value is a main technical parameter to describe the impact of each input variable on the response value. In short, the larger F value, the more significant the corresponding variable is. From the Table 3, the F value of polynomial regression model is 2567.59, far exceeding the F-test critical value F_0.05_ (9, 7) = 3.677, which means the developed model is significant. Meanwhile, the value of ‘Prob > F’ is less than 0.05 meaning the corresponding model term is significant. So, in this model, *T*, *F*, *P*, *TF*, *TP*, *FP*, *T*^2^, *F*^2^ and *P*^2^ are all the significant model terms. Here, *T*, *F* and *P* represents the heating temperature, tape tension and roller pressure, respectively. At last, the ‘Prob > F’ value of ‘Lack of Fit’, 0.2749, means the lack of fit terms were not significant relative to the pure error. Therefore, the corresponding model can be considered a complete model including all the necessary items.

**Figure 3:**
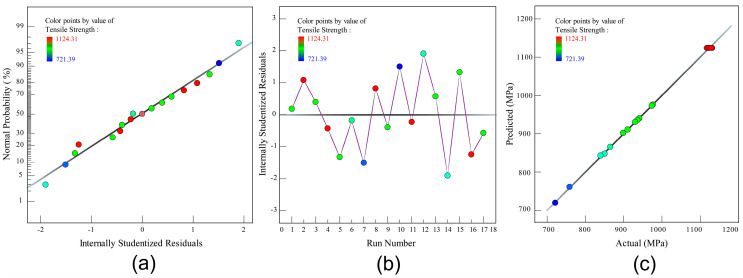
Residual analysis results. (**a**) Residuals normal plot of tensile strength model; (**b**) Residual versus run plot of tensile strength model; (**c**) Predicted versus actual plot of tensile strength model.

In addition, the residual analysis had been employed to validate the reliability of corresponding polynomial regression model. Figure 3a shows the diagram of internally studentized residuals. In the graph, the residuals scatters are an approximate linear distribution along a straight line. The result meets the requirement of normal distribution meaning that the fitted model for tensile strength is trustworthy. According to the residual operation diagram in Figure 3b, the residuals scatters is closer to the random distribution pattern. Meanwhile, Figure 3c gives the comparison between the actual and predicted value of the tensile strength. The max error between the actual and predicted value is less than 5%. Therefore, the developed polynomial regression model can be considered as reasonable for the composite prepreg tape winding process. In brief, the established second order polynomial regression model could be effectively utilized to predict the tensile strength of winding products.

## 4. Influence Mechanism and Optimization of Parameters

### 4.1. Sensitivity Analysis for Single Parameter

Parameters sensitivity denotes the sensitive degree of target value to each design variable change [25,26]. In this study, sensitivity analysis for single parameter were applied to make clear the influence mechanism of each winding parameter changes on tensile strength. In a mathematical sense, the sensitivity coefficient is the first-order derivative of model output with reference to the model parameter [41]. Therefore, let *S*(*x_i_*) denote the sensitivity coefficients, we can obtain the sensitivity of single parameter as follows:

(5) $$S\left(x_{i}\right)=\underset{\Delta x_{i}\to 0}{lim}\frac{f\left(x_{i}+\Delta x_{i}\right)-f\left(x_{i}\right)}{\Delta x_{i}}=\frac{\partial f\left(x\right)}{\partial x_{i}}$$

where *S*(*x_i_*) denotes the sensitivity coefficients, *f*(*x*) is the model output and *x_i_* (*i* = 1, 2, 3, …, n) is the *i*th input parameter.

In the experiment, the tensile strength model was fitted by a battery of discrete experimental data. To obtain a given single factor model, the other two factors were simultaneously set as the zero level. The sub models of heating temperature, tape tension and roller pressure are as follows,

(6) $$\begin{array}{c} y_{T}=f\left(x_{1},{\overset{¯}{x}}_{2},{\overset{¯}{x}}_{3}\right)=1121.008+10.31\cdot x_{1}-71.604\cdot x_{1}{}^{2}\\ y_{F}=f\left({\overset{¯}{x}}_{1},x_{2},{\overset{¯}{x}}_{3}\right)=1121.008+12.488\cdot x_{2}-182.739\cdot x_{2}{}^{2}\\ y_{P}=f\left({\overset{¯}{x}}_{1},{\overset{¯}{x}}_{2},x_{3}\right)=1121.008+8.758\cdot x_{3}-101.6889\cdot x_{3}{}^{2} \end{array}$$

where the *y_T_*, *y_F_* and *y_P_* denote the sub models of heating temperature, tape tension and roller pressure, respectively; and ${\overset{¯}{x}}_{1}$, ${\overset{¯}{x}}_{2}$ and ${\overset{¯}{x}}_{3}$ represent the zero level of each variable.

According to Equation (5), the sensitivity models of three winding parameter including heating temperature, tape tension and roller pressure can be described as follows:

(7) $$\begin{array}{c} y_{T}^{’}=\partial f\left(x_{1},{\overset{¯}{x}}_{2},{\overset{¯}{x}}_{3}\right)/\partial x_{1}=10.31-143.208\cdot x_{1}\\ y_{F}^{’}=\partial f\left({\overset{¯}{x}}_{1},x_{2},{\overset{¯}{x}}_{3}\right)/\partial x_{2}=12.488-365.478\cdot x_{2}\\ y_{P}^{’}=\partial f\left({\overset{¯}{x}}_{1},{\overset{¯}{x}}_{2},x_{3}\right)/\partial x_{3}=8.758-203.3778\cdot x_{3} \end{array}$$

**Figure 4:**
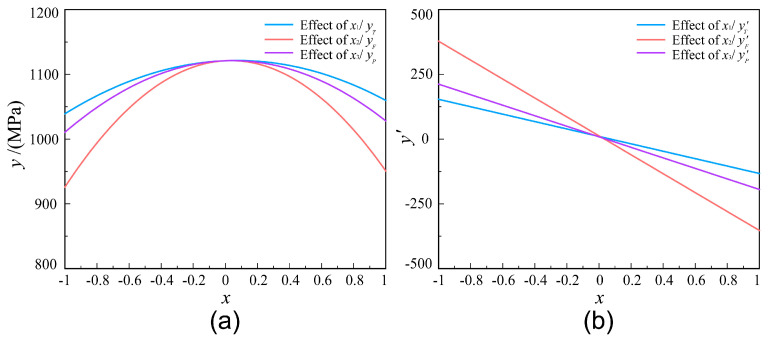
Effects of processing parameters. (**a**) The regression curve of sub-model; (**b**) The sensitivity model of each factor.

Figure 4 shows the effects of processing parameters on the tensile strength model. As shown in Figure 4a, the regression curves of sub-model are parabola going downwards. The results show that the tensile strength increases first and then decreases with the increase of each process parameter. Meanwhile, *x*_2_ has significant effect on the tensile strength of tape winding products. The value of tensile strength will change greatly as the value of *x*_2_ changes. The tensile strength, relatively speaking, are not so sensitive to the *x*_1_ and *x*_3_. According to Figure 4b, the images of sensitivity model of each factor are three linear functions with different slopes. The results show that, for the composite tape winding process, the tensile strength is most sensitive to the variation of tape tension, next sensitive to roller pressure and least sensitive to heating temperature. All the process parameters have a positive impact first and then a negative one on the tensile strength. In addition, the impact degree is gradually weakened with the increasing of the variable first and then gradually strengthened.

### 4.2. Iso-Surfaces Analysis for Parameter Range

The previous sensitivity analysis shows that the tensile strength of composite winding products is deeply influenced by the processing parameters. In three-dimensional space, a set of curved surface graphs are fitted by points with the same magnitude in a certain physical quantity. Then the curved surface can be applied to describe the distribution law of those physical quantities with continuous distribution characteristics [28].

**Figure 5:**
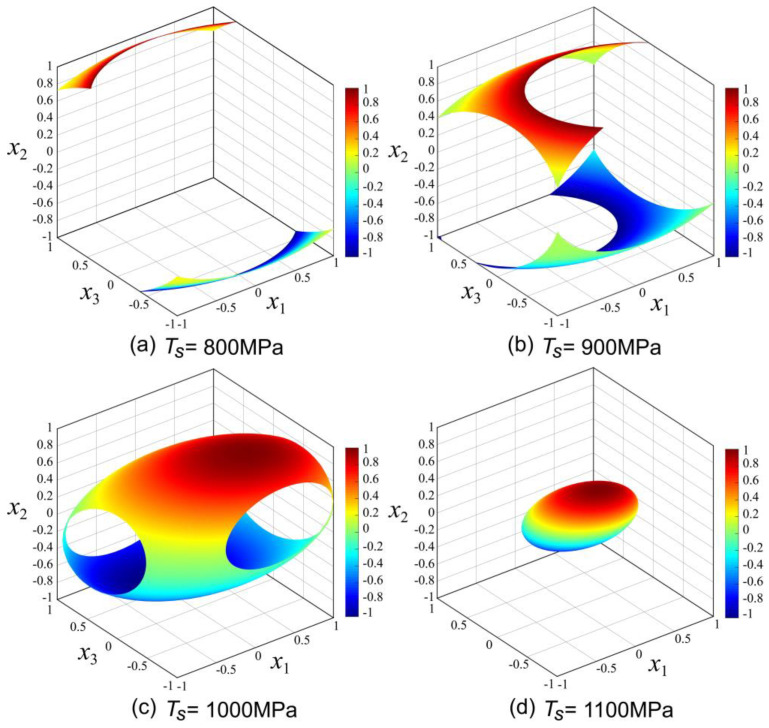
Iso-surfaces for different tensile strength values in three-dimensional factor space. (**a**) Iso-surface diagram of *T_S_* = 800 MPa; (**b**) Iso-surface diagram of *T_S_* = 900 MPa; (**c**) Iso-surface diagram of *T_S_* = 1000 MPa; (**d**) Iso-surface diagram of *T_S_* = 1100 MPa.

According to the experiment’s results, the range of tensile strength value is 721.39~1124.31 MPa. For a more intuitive analysis of process parameters, four representative tensile strength values 800, 900, 1000 and 1100 MPa were selected as the certain objective value to complete drawing of three-dimensional iso-surfaces. The iso-surfaces analysis of different tensile strength values in Equation (4) were carried out on MATLAB (R2017a, The MathWorks Company, Natick, MA, USA) software platform. The results of iso-surfaces analysis are displayed in the Figure 5a–d. For the iso-surfaces of *T_s_* = 800, 900 and 1000 MPa, *x*_1_ has the same range of [−1, 1] as shown in Figure 5a–c. Only in the case of *T_s_* = 1100 MPa in Figure 5d, *x*_1_ falls into the [−0.49, 0.64]. When the tensile strength value is 800 MPa, *x*_2_ has the compound interval [−1, −0.54] and [0.72, 1] in Figure 5a. Similarly, *x*_2_ has a compound interval of [−1, −0.02] and [0.33,1] when *T_s_* = 900 MPa in Figure 5b. As shown in Figure 5c,d, *x*_2_ falls into the [−0.85, 0.92] and [−0.34, 0.41], respectively. For the iso-surfaces of *T_s_* = 900 and 1000 MPa, *x*_3_ has the same interval [−1, 1] in Figure 5b,c. However, in Figure 5c, *x*_3_ falls into the compound interval [−1, −0.37] and [0.45, 1] when *T*_s_ = 800 MPa. In addition, in Figure 5d, *x*_3_ falls into the range of [−0.49, 0.52]. The iso-surfaces analysis indicate that in the composite tape winding process, for a particular value of *T_s_*, the parameter interval of *x*_2_ is the narrowest, followed by *x*_3_ and *x*_1_. Meanwhile, the results indicate that *x*_2_ has the largest impact on the value of *T*_s_. Therefore, to get a higher tensile strength value, the parameter interval and control strategy of *x*_2_ need to be seriously considered.

### 4.3. Coupling Analysis for Process Parameters

In the actual winding process of composite materials prepreg tape, individual process parameters have a crucial impact on the performance of the winding product. In addition, the coupling between various process parameters will have a non-negligible interactive effect on the quality of the product such as tensile strength. The influence mechanism of each process parameter on tensile strength is affected by other parameters value. The process parameters including heating temperature, tape tension and winding pressure are coupled with each other to determine the final tensile strength of winding product. According to the response surface analysis results, the polynomial regression model of the tensile strength in composite tape winding process can be obtained as:

(8) $$\begin{array}{cc} T_{S}=f\left(T,F,P\right)= & -290.644+11.903T+3.356F+0.741P+3.592\times 10^{-3}T\cdot F-1.199\times 10^{-3}T\\ & \cdot P-6.435\times 10^{-4}F\cdot P-7.956\times 10^{-2}T^{2}-4.568\times 10^{-3}F^{2}-1.808\times 10^{-4}P^{2} \end{array}$$

where *T_S_* represents the tensile strength of winding product, MPa; *T* denotes the heating temperature, °C; *F* is the tape tension, N; and *P* is the roller pressure, N.

**Figure 6:**
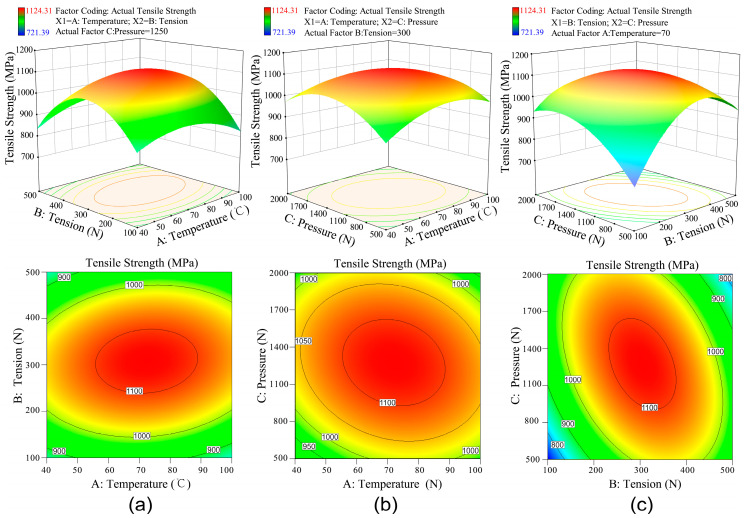
The response surface and contour lines map of process parameters on the tensile strength. (**a**) Coupling effect of temperature and tension; (**b**) Coupling effect of temperature and pressure; (**c**) Coupling effect of tension and pressure.

The coupling effect of process parameters on the tensile strength of winding products were analyzed using the response surface design software Design Expert 10 (Stat-Ease, Inc., Minneapolis, MN, USA). And Figure 6 shows the response surfaces and contour lines for the effect of parameters coupling on tensile strength. Figure 6a demonstrates the coupling effect of heating temperature and tape tension on the tensile strength of winding product. During the winding molding process, prepreg tapes can be heated to enhance the viscous fluidity of the resin. The heated resin is conducive to improving the degree of interface bonding between the resin and the fiber and between the tape and the laminate. When the temperature is low, the resin has poor fluidity. At this time, a small tape tension cannot provide a suitable deformation for the prepreg tape. However, excessive tension can easily cause the breaking of fibers in the tape, resulting in uneven stress distribution. When the temperature is too high, the resin will undergo a local curing reaction, which will reduce the overall mechanical properties of the winding product. The highest value of tensile strength locates in the area of 55~85 °C for heating temperature and 250~350 N for tape tension.

Figure 6b displays the coupling effect of heating temperature and roller pressure on the tensile strength of winding product. The positive pressure produced by the compaction roller can be employed to squeeze out the bubbles between the tape and laminated layers. The resin on the contact surface of the prepreg tape and the laminate will fuse into one under the suitable temperature and positive pressure. When the temperature is low, the polymer matrix does not reach the molten state. At this moment, a large pressure is difficult to improve the surface bonding strength for the poor degree of molecular chain diffusion. With the increase of temperature, the tensile strengths of the winding products had improved significantly. However, an excessive high temperature will cause the curing reaction of the polymer matrix, which will seriously reduce the products’ mechanical properties. When the temperature is suitable, too small pressure cannot make the tape and the laminate come into intimate contact. Conversely, extreme high positive pressure will excessively extrude the resin in a molten state from the laminates in large amounts. Looking at the whole picture, the tensile strength has a generally high value. As shown in Figure 6b, the ideal tensile strength appears in the area of 55~85 °C for heating temperature and 1000~1500 N for roller pressure.

Figure 6c shows the coupling effect of tape tension and roller pressure on the tensile strength of winding product. As a whole, the span of the tensile strength range is relatively large. The lager tensile strength values appear in the area of 250~350 N for tape tension and 900~1600 N for roller pressure. When both the tape tension and roller pressure are very low or high, the value of tensile strength is the smallest. Meanwhile, the value of tensile strength will first increase rapidly and then decrease greatly with the increase of the tape tension. When the tape tension is small, a large number of gaps appear between the tape and the base laminate. If the roller pressure is also small, at this time, these bubbles will be difficult to squeeze out. The degree of bonding between the winding layers becomes lower, resulting in a significant decrease for tensile strength. When the tape is wound around the mandrel through the hot pressing roller, the tension will generate a part of the normal pressure. If the tension and pressure are both large, the tape will be severely deformed, which will severely affect the mechanical properties of the winding product.

### 4.4. Optimization and Validation

To obtain a greater tensile strength value, the excellent parameter combination needs to be provided by optimizing process based on the desirability function. In this paper, the response was optimized using the general linear scale desirability function. The desirability function was beneficial for searching for the most favorable point in the design space [34,42]. Here, the response of tensile strength was the larger the better. Let *ŷ* represents the target experimental response. When the *ŷ* exceeds a particular criteria value, the desirability value equals to 1. If the *ŷ* is less than a particular criteria value, the desirability value equals to 0. Then the desirability function for the response to be maximized can be described as follow [35]:

(9) $$d_{i}=\{\begin{array}{cc} 0, & \;\hat{y}\leq y_{min}\\ {\left(\frac{\hat{y}-y_{min}}{y_{max}-y_{min}}\right)}^{r}, & \;y_{min}\leq \hat{y}\leq y_{max}\\ 1, & \;\hat{y}\geq y_{min} \end{array}$$

where *d_i_* is the individual desirability of the response to be maximized; *y_min_* and *y_max_* denote the lowest and highest limits for each response, respectively; and *r* represents the weight, which can be specified by the user, *r* ≥ 0.

A global desirability function, *D*, also be called as overall desirability, is defined as the geometric mean of individual desirability values in Equation (10) [30]:

(10) $$D={\left(\overset{n}{\underset{i=1}{\prod }}d_{i}\right)}^{1/n}$$

where the *n* is the number of the experimental responses.

In this article, the response surface design software Design Expert 10 (Stat-Ease, Inc., Minneapolis, MN, USA) was applied to determine the maximum desirability. The results were sorted by the desirability value from largest to smallest. Then the combination of process condition with a highest desirability value *D* will be selected as the optimum condition for the response.

**Figure 7:**
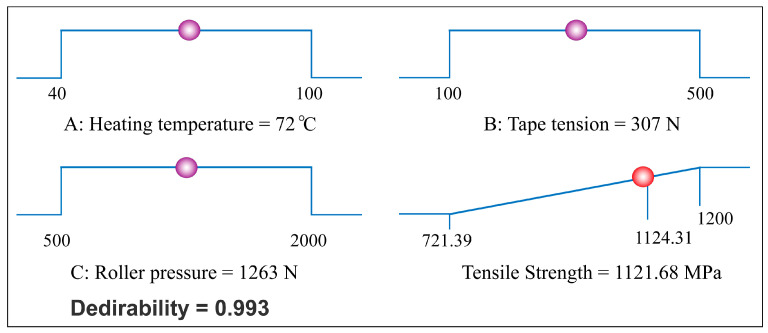
Ramp function graph of desirability and the optimized result for tensile strength.

According to the analysis results of the response surface software, the optimal parameter combination was selected as heating temperature with 72 °C, tape tension with 307 N and roller pressure with 1263 N. The maximum tensile strength value predicted by the response surface model was 1121.68 MPa. Figure 7 shows the desirability values for the response value. As shown in Figure 7, the highlighted dot from each ramp represents the recommended value for corresponding process parameter. Meanwhile, the desirability value was 0.993, which means that the target value and the response value have a high degree of closeness. To verify the effectiveness and reliability of the optimization results, three groups of repeated winding experiments were carried out using the same developed parameter combination. The materials, environment, testing process and so forth, in the verification experiments were the same as the previous experiments. Table 4 shows the results of validation experiments using the optimal parameter combination. The average tensile strength value of the testing specimens, 1131.26 MPa, was 9.58 MPa higher than the predicted value. Meanwhile, the average relative error of the verification tests was 0.85%. The comparison results showed that the quadratic regression model provided by response surface methodology was useful and reliable for the prepreg tape winding process. In addition, the optimal parameter combination has a positive guiding significance for improving the quality of winding products.

## 5. Conclusions

This article was dedicated to researching the influence mechanism and optimization analysis of parameters for the composite prepreg tape winding process. Process parameters including heating temperature, tape tension and roller pressure have an important impact on the winding products’ mechanical property such as tensile strength.

According to sensitivity analysis for single parameter, the tensile strength is most sensitive to the variation of tape tension, next sensitive to roller pressure and least sensitive to heating temperature. The tape tension has the most significant effect on the tensile strength of winding products. Meanwhile, the parameter interval of the processing parameters can be effectively determined via iso-surfaces for different tensile strength values in three-dimensional factor space. For a particular value of tensile strength, the parameter interval of tape tension is the narrowest according to the iso-surface diagrams of tensile strength. When only considering the enhancement of the tensile strength, the parameter interval and control strategy of tape tension needs to be confirmed first. In addition, three key process parameters, including heating temperature, tape tension and roller pressure, are coupled with each other to determine the tensile strength of the winding products. Then the outstanding parameter combination with the heating temperature of 72 °C, tape tension of 307 N and roller pressure of 1263 N was provided by response surface design software via desirability function method. The validation experiments showed that the optimal parameter combination has a positive guiding significance for improving the quality of the winding product.

## Figures and Tables

**Table 1:** Level of process parameters.

				Levels		
Experimental Parameters	Symbol	Code	Units	Level −1	Level 0	Level 1
Heating temperature	*T*	*x*_1_	°C	40	70	100
Tape tension	*F*	*x*_2_	N	100	300	500
Roller pressure	*P*	*x*_3_	N	500	1250	2000

**Table 2:** The experiment results.

No.	*T*/(°C)	*F*/(N)	*P*/(N)	*x*_1_	*x*_2_	*x*_3_	*T_S_*/(MPa)
1	100	500	1250	1	1	0	911.33
2	70	300	1250	0	0	0	1124.31
3	100	300	500	1	0	−1	976.92
4	70	300	1250	0	0	0	1119.68
5	40	300	500	−1	0	−1	899.4
6	40	100	1250	−1	−1	0	865.11
7	70	500	2000	0	1	1	758.72
8	70	300	1250	0	0	0	1123.53
9	40	300	2000	−1	0	1	972.47
10	70	100	500	0	−1	−1	721.39
11	70	300	1250	0	0	0	1120.32
12	40	500	1250	−1	1	0	850.54
13	70	100	2000	0	−1	1	930.36
14	100	100	1250	1	−1	0	839.68
15	100	300	2000	1	0	1	942.07
16	70	300	1250	0	0	0	1117.2
17	70	500	500	0	1	−1	935.85

**Table 3:** ANOVA results for response surface quadratic model of tensile strength.

Source	SS	DF	MS	*F* Value	Prob > *F*
Model	2.7 × 10^5^	9	30,002.31	2567.59	<0.0001
*T*	850.37	1	850.37	72.77	<0.0001
*F*	1247.5	1	1247.5	106.76	<0.0001
*P*	613.55	1	613.55	52.51	0.0002
*TF*	1858.47	1	1858.47	159.05	<0.0001
*TP*	2911.68	1	2911.68	249.18	<0.0001
*FP*	37,268.3	1	37,268.3	3189.41	<0.0001
*T* ^2^	21,587.93	1	21,587.93	1847.49	<0.0001
*F* ^2^	1.406 × 10^5^	1	1.41 × 10^5^	12,032.87	<0.0001
*P* ^2^	43,539.59	1	43,539.59	3726.1	<0.0001
Residual	81.8	7	11.69		
Lack of Fit	47.79	3	15.93	1.87	0.2749
Pure Error	34	4	8.5		
Cor Total	2.701 × 10^5^	16			
DF: Degrees of Freedom; SS: Sum of Squares; MS: Mean Square					

**Table 4:** Results of validation experiments using the optimal parameter combination.

**No.**	***T*/(°C** **)**	***F*/** **(N** **)**	***P*/(N)**	**Tensile Strength/(MPa)**		**Relative Error**
				**Predicted**	**Experiment**	
1	72	307	1263	1121.68	1129.16	0.67%
2					1133.83	1.08%
3					1130.79	0.81%
